# Self-regulated learning constructs as predictors of academic performance among Saudi EFL students in virtual learning environments: Insights from self-regulated learning theory

**DOI:** 10.3389/fpsyg.2026.1859492

**Published:** 2026-07-21

**Authors:** Ayman Khafaga, Mohammed Muflih Alamer

**Affiliations:** 1 Department of English Language, College of Science & Humanities, Prince Sattam bin Abdulaziz University, Al-Kharj, Saudi Arabia; 2 Department of English Language, Faculty of Arts & Humanities, Suez Canal University, Ismailia, Egypt; 3 Department of Curriculum & Instruction, College of Education, King Saud University, Riyadh, Saudi Arabia

**Keywords:** academic performance, goal setting, Saudi EFL context, self-evaluation, self-monitoring, self-regulated learning theory, time management, virtual learning

## Abstract

Drawing on Zimmerman’s (1989) self-regulated learning (SRL) theory, this paper investigates how self-regulated learning constructs are associated with and predict academic performance among Saudi EFL learners within virtual learning environments. More specifically, it scrutinizes how goal setting, time management, self-monitoring, and self-evaluation predict academic performance in virtual EFL contexts. A quantitative approach was adopted, constituting a questionnaire administered to 270 students. Findings reveal that SRL constructs have potential in terms of being predictors of academic performance among Saudi EFL majors in virtual learning settings; and in this case, self-monitoring and self-evaluation have been the most effective predictors. Moreover, it is also observed that reflective behaviors and proactive attitudes on the part of students are significant correlates of Saudi EFL majors’ academic performance in virtual learning environments (VLEs). The educational suggestions derived from these results include the necessity to incorporate SRL-related programs into the virtual EFL classes as well as solve students’ metacognition and time management problems. Such recommendations are valuable for the design of SRL-promoting virtual EFL environments in Saudi Arabia.

## 1 Introduction

Virtual learning has developed as an important teaching and learning tool in the arena of higher education institutions with the development and spread of virtual learning platforms and the global implementation of virtual learning environments (VLEs) (Almayez et al., 2025). Within the scope of English as a foreign language (EFL) learning, the use of virtual learning has enabled learners to attain the advantage of flexibility and convenience in self-learning; however, it has also compelled learners to assume the responsibility of the regulation of their own learning processes. In this regard, the concept of “self-regulated learning” (SRL) has attracted considerable attention as a theoretical model in the exploration of the learning processes of learners (Jaramillo et al., 2022; García-Ros et al., 2023). According to SRL theory, the process of learning is not only the acquisition of knowledge, but rather a cyclical process wherein learners are the active participants in the setting of goals and the evaluation of the outcome (Zimmerman, 2000; Pintrich, 2000; Lourenço and Paiva, 2024). Such processes are specifically important in VLEs, where learners need to monitor their learning behavior in the absence of instructors’ or peers’ continuous physical presence. Previous studies have indicated that SRL constructs, such as goal-setting, time management, self-monitoring, and self-evaluation, are associated with autonomy and engagement in VLEs (Hunutlu, 2023; Chan et al., 2026).

In EFL learning contexts, particularly in countries where English is taught as a foreign language such as Saudi Arabia, the transition to VLEs has introduced new pedagogical opportunities as well as challenges. Saudi universities have increasingly adopted virtual learning platforms, such as the Blackboard Collaborate, to support language instruction, enabling students to access resources, communicate with instructors, and complete learning tasks remotely (Alreshoud and Abdelhalim, 2022). Yet, it can be assumed that the VLEs usually expect greater levels of self-management from students. Students’ inability to regulate their own behavior could cause problems for them when they face distractions, inefficient learning strategies, or negative outcomes of their study efforts (Alanazi, 2020). According to the findings in previous studies (e.g., Tao et al., 2025; Zhao et al., 2025; Alhroob and Al-khresheh, 2026), self-regulated learners demonstrate improved planning, monitoring, and regulating skills, resulting in enhanced academic performance. Within EFL contexts, these strategies are related to better learning outcomes and academic performance.

Nevertheless, despite the emergence of virtual learning in the higher education sector of Saudi Arabia, some obstacles are evident with regard to the capacity of the learner to learn independently. In the context of SRL, the main reasons for the difficulties faced by the learners in terms of independent learning can be identified as the inability of the learners to cultivate their intrinsic potential to regulate their own learning (Shamla and Jayan, 2025). According to Zimmerman (2002), goal setting plays an essential role in assisting the learner in setting personal objectives and guiding them to achieve their linguistic competence. He maintains that time management plays a key role in ensuring that the learner allocates adequate time for learning and practicing linguistic skills, as well as completing assignments. As for self-monitoring, it assists the learner in monitoring their personal progress, which enables them to assess their strengths and weaknesses. Concerning self-evaluation, it allows the learner to review their progress and the efficiency of the learning strategies used. Research into language learning has shown that these strategies interact dynamically, demonstrating significant correlations of students’ engagement and their motivation to study language (Zimmerman, 2002; Schunk and Zimmerman, 2008; Baissane and Zaid, 2025; Al-Nafjan et al., 2025).

While there are numerous investigations into self-regulated learning in terms of general educational processes (e.g., Yot-Dominguez and Marcelo, 2017; Sutarni et al., 2021; Brenner, 2022; Ucar, 2023; Arrafii et al., 2025), the issue of what particular features of self-regulated learning have associations with the academic performance of Saudi EFL students when studying in a fully virtual environment is still worthy of linguistic research. To clarify, many previous studies are dedicated to examining the general issue of self-regulation learning rather than to the problem of the extent to which individual constructs of self-regulated learning, particularly within VLEs. Moreover, empirical studies dedicated to investigating how goal-setting, time management, self-monitoring, and self-evaluation are associated with academic performance of Saudi EFL students at university level remain rare (Alotaibi et al., 2017; Alanazi, 2020). Arguing from this context, this study endeavors to investigate the connection between virtual learning and the academic performance of Saudi EFL students in relation to the SRL theory. More specifically, this study explores how self-regulated learning constructs of goal setting, time management, self-monitoring, and self-evaluation are associated with and predict academic performance among Saudi EFL learners within VLEs. As such, this study seeks to answer the following overarching research question:

*RQ1*: To what extent do the four self-regulated learning constructs (goal setting, time management, self-monitoring, and self-evaluation) are significant predictors of Saudi EFL majors’ academic performance in VLEs?

The answer to this overarching research question comprises the main objectives this study aims to achieve: to explore the extent to which self-regulated learning constructs, namely goal setting, time management, self-monitoring, and self-evaluation, are significant correlates of academic performance among Saudi EFL majors within VLEs. Arguing from this context, the following hypotheses are proposed:

*H1*: Goal setting is significantly associated with academic performance of Saudi EFL majors in VLEs.

*H2*: Time management is significantly correlated with academic performance of Saudi EFL majors in VLEs.

*H3*: Self-monitoring has positive correlations with academic performance of Saudi EFL majors in VLEs.

*H4*: Self-evaluation has (a) significant relationship with academic performance of Saudi EFL majors in VLEs.

## 2 Literature review

Virtual learning has been identified as one of the essential features of higher learning institutions across the globe, particularly in language learning contexts. VLEs in EFL contexts enable learners to access learning, engage with interactive learning activities, and communicate with the teacher beyond physical classroom walls (Hockly, 2015). However, the academic performance of EFL learners through VLEs depends, in most cases, on learners’ ability to self-regulate their learning behavior, particularly their engagement with learning activities provided by the learning environment. While in face-to-face instruction, teachers play a crucial role in facilitating learners’ learning, in VLEs, learners are required to self-regulate their learning by managing their learning time, learning progress, and learning outcomes. As a result, EFL learners without the ability to self-regulate their learning behavior cannot benefit from learning through VLEs as expected (Broadbent and Poon, 2015).

The self-regulation learning (SRL) theory offers an extensive framework for understanding how learners manage their learning processes in the classroom. As Zimmerman (1989, 2002) argues, the self-regulated learning approach considers the learners as active participants in the learning process, as they set learning goals, select learning strategies, monitor, and evaluate learning processes to accomplish the desired learning outcomes. Pintrich (2004) also perceives SRL theory as an iterative process wherein learners plan, monitor, and reflect on the learning processes, as they continue to adjust the learning strategies in light of the feedback and performance received in the learning process. The SRL proves useful in VLEs, as the learners need to manage the learning processes independently without the constant guidance of the instructor. Several studies (e.g., Dignath and Büttner, 2008; Hunutlu, 2023; Simón-Grábalos et al., 2025; Seyyedi, 2026) confirm that the use of the SRL theoretical principles in the learning processes helps learners to achieve better learning outcomes, increased motivation, and persistence in the learning processes. These studies also emphasize that within the context of language learning the application of the SRL approach helps learners to achieve vocabulary, reading, and writing learning outcomes as they enthusiastically participate in the learning processes.

Furthermore, among the essential elements of self-regulated learning, “goal setting” is of critical importance for guiding students’ learning behaviors. According to Zimmerman (2002), “goal setting” refers to the process by which individuals establish specific goals and strategies for attaining those goals. Locke and Latham (2002) argue that specific and challenging goals facilitate motivation and help students’ attention focus on specific and related learning activities. In EFL learning settings, for example, students’ goal setting include setting specific objectives for developing their vocabulary, reading, or communicative competence. Empirical research (e.g., Fatmala, 2025; Tao et al., 2025; von Keyserlingk et al., 2026) has demonstrated that students’ setting of particular and clear academic goals improves their probabilities of effectively organizing their learning activities and persisting through difficulties. These studies also argue that in VLEs, students’ goal setting is of critical importance for helping students effectively organize their independent learning activities and avoid distractions. This, in turn, indicates that students’ goal setting is positively correlated with students’ engagement with virtual language learning activities (Zhao et al., 2025).

The other important SRL skill that plays a significant role in students’ academic performance is “time management,” which involves learners’ ability to make appropriate allocations of time for learning activities (Zimmerman and Schunk, 2011). In VLEs, students need to make appropriate allocations of time to balance their learning activities because they may face difficulties in managing their time due to procrastination, distractions, and irregularities in learning habits. Previous studies (e.g., Broadbent and Poon, 2015; Barnard et al., 2009; Zhang, 2024) indicate that students who poorly manage their learning time may experience negative impacts on their academic performance. They maintain that students who poorly manage their learning time may become less engaged in virtual learning environments. In EFL learning contexts, students who make appropriate allocations of time may assign adequate time to practice the various language skills. As such, students who make appropriate allocations of time become more engaged in VLEs, and their academic performance is likely to be improved (Waheed et al., 2025).

Besides goal-setting and time management, self-monitoring and self-evaluation are other cognitive and metacognitive processes of self-regulated learning. Self-monitoring occurs when students observe their own learning process and check their understanding of the learning tasks they are undertaking (Zimmerman, 2002). In EFL learning, this could mean checking their comprehension of the texts they are reading, checking their written assignments, or evaluating their performance in the language learning tasks they are undertaking. Self-evaluation, on the other hand, is when students review what they have learned and evaluate whether they have accomplished their learning goals (Zimmerman, 2002). According to Nicol and Macfarlane-Dick (2006), self-evaluation helps students improve their critical thinking and reflective skills, which are necessary to improve their learning continuously. In virtual learning, self-monitoring and self-evaluation help students evaluate their own learning and regulate their learning strategies as appropriate. Many previous studies come to terms that students who engage in self-monitoring and self-evaluation often demonstrate higher levels of academic performance and become independent learners than those who engaged in these practices less frequently (Panadero, 2017; Gambo and Shakir, 2021).

The selection of goal-setting, time management, self-monitoring, and self-evaluation stemmed from Zimmerman (2002) cyclical model of self-regulated learning. In light of the cyclical model of SRL, the process of learning involves a cyclical process involving consideration, performance, and self-reflection components. Goal-setting and time management involve planning processes, which entail setting objectives and organizing resources prior to engaging in a learning task. Self-monitoring requires performance processes, wherein learners evaluate how much they have learned during an activity. Self-evaluation constitutes the self-reflection component of the learning process, in which learners evaluate their learning processes in light of achieving the desired goals. These four processes cover all the major self-regulatory processes suggested by Zimmerman (2002).

Although a substantial body of research has established positive associations between SRL and academic performance, much of the existing literature has examined SRL either as a global construct or within traditional educational settings (e.g., Waheed et al., 2025; Zheng and Sun, 2025). Comparatively less attention has been devoted to understanding how specific SRL constructs operate within VLEs, particularly in Saudi EFL context. Furthermore, previous studies have often focused on confirming the general relationship between SRL and academic outcomes rather than examining the relative correlations of distinct regulatory processes across the phases of Zimmerman (2002) cyclical model. As virtual learning requires learners to assume greater responsibility for managing their learning, monitoring their progress, and evaluating their performance, it remains important to investigate whether certain SRL constructs are more strongly associated with academic performance than others. Examining these relationships within the Saudi EFL context may therefore offer a much more understanding of how SRL functions in VLEs and whether existing theoretical assumptions regarding the role of SRL can be generalized across different cultural, linguistic, and educational settings.

In the Saudi education sector, virtual learning has gained immense popularity. The use of learning management tools, particularly the Blackboard Collaborate platform, has a significant correlation with this increase and is clearly manifested in EFL learning. This has allowed students the opportunity to access their learning materials and participate in discussions from anywhere (Alhousayen and Alghammas, 2025). This has also created an urgent need for students to be equipped with SRL abilities. In this regard, previous research has shown that there is a significant relationship between virtual learning and students’ ability to manage their own learning (Alreshoud and Abdelhalim, 2022; Alhousayen and Alghammas, 2025; Almayez et al., 2025). On the other hand, other studies have shown that EFL students lack the ability to motivate themselves and assess their performance in a virtual setting (Tomak and Seferoğlu, 2021; Noori, 2025).

Another significant aspect of SRL in the context of virtual EFL instruction is the relationship that SRL constructs have with academic performance. When we think about measuring the achievement of second language learners, we tend to think in terms of clear and identifiable measures, such as course grades, language proficiency tests, assignment completion, etc. (Zimmerman and Schunk, 2011). The literature has consistently demonstrated that when SRL strategies are utilized by second language learners, they are more likely to achieve academic goals than learners who rely on more traditional, passive approaches to instruction (Alanazi, 2020; Waheed et al., 2025; Zheng and Sun, 2025). In the context of virtual learning, SRL strategies are particularly significant because they are a part of the student’s self-directed learning process. Zimmerman (2000) argue that metacognitive SRL strategies, particularly with regards to time management, effort regulation, and self-evaluation, are considerably linked to academic achievement in the context of virtual learning. SRL strategies with regards to second language instruction enable learners to engage in regular practice, monitor their own achievement, and adjust their strategies when confronted with difficulties (Faza and Lestari, 2025). As such, self-regulated second-language learners are anticipated to show better levels of persistence, engagement, and academic performance in virtual EFL contexts.

Moreover, recent research (e.g., Panadero, 2017; Zhang, 2024; Zhao et al., 2025) emphasizes the significance of metacognitive awareness and learner autonomy in enhancing self-regulation in digital learning. In this regard, metacognitive awareness allows students to monitor their learning strategies and assess their effectiveness in achieving their learning goals. In the case of EFL learning, metacognitive awareness involves students’ awareness of their struggles in understanding grammatical structures, remembering vocabulary, and writing. In such cases, students may use different strategies to improve their learning. According to Barnard et al. (2009), students with high metacognitive awareness are more adaptive and effective in coping with VLEs and their academic demands. For Almayez et al. (2025), in the Saudi higher educational context, the promotion of SRL strategies and metacognitive awareness is becoming critical with the continuous expansion of virtual learning initiatives in universities in line with educational modernization. SRL strategies may be effectively incorporated into VLEs to support Saudi EFL students’ independence and self-regulatory practices.

## 3 Methodology

### 3.1 Design of the study

This study adopted a quantitative method to investigate the relationship between virtual learning and the academic performance of Saudi EFL majors by drawing on Zimmerman (1989) SRL theory. Quantitative methods are widely used in educational studies because they are effective in testing the relationship between variables and validating theories (Creswell and Creswell, 2018). In this study, the components of self-regulated learning, namely goal setting, time management, self-monitoring, and self-evaluation, are considered as the independent variables, while the academic performance of the EFL students in their virtual EFL courses is considered as the dependent variable. Academic performance was operationalized as students’ cumulative GPA on a 4-point scale, which was self-reported by participants at the time of questionnaire administration. GPA was used as a widely recognized indicator of academic achievement in higher education and served as the outcome variable in examining the relationships between self-regulated learning constructs and academic performance. The quantitative method adopted in this study was the survey correlational method, which is appropriate for investigating the relationship between changes in SRL strategies and their outcomes in a virtual environment. This method is particularly appropriate in studying the perceptions, behaviors, and strategies of learners in a higher education setting (Dörnyei, 2007).

### 3.2 Participants

The study participants included undergraduate Saudi students studying at Prince Sattam bin Abdulaziz University, Saudi Arabia, wherein courses in EFL using virtual learning management systems to teach language skills were offered. Convenience sampling was used to recruit participants from the selected university, where Blackboard Collaborate was primarily used as the main digital platform for delivering all virtual EFL courses. The participants included 270 EFL students; 165 are males and 105 are females. They were from different academic levels (from level 1 to level 8), with varying learning experiences and academic performance. All the participants had prior experience using the virtual learning environment with virtual lectures, virtual assignments, and virtual discussion forums. All the participants’ rights and ethical considerations were observed throughout the study by adhering to the ethics of conducting educational research (Cohen et al., 2018).

### 3.3 Data collection and instrument

The researchers utilized a structured questionnaire to determine the extent of the students’ regulation of learning in the virtual courses they were taking. The tool was supposed to be appropriate as it was built upon the current research base for the study of online self-regulated learning that includes the use of the Online Self-Regulated Learning Questionnaire (OSRLQ) by Barnard et al. (2009), who have successfully validated its use within the scope of virtual learning research. In addition to the use of the questionnaire, the researchers employed a Likert scale from strongly disagree to strongly agree in order to evaluate the students’ regulation of their learning, including their approaches towards learning, such as setting learning goals, managing time and assessing themselves. The researchers also collected demographic data regarding the gender, level and prior experience of using virtual learning tools of the respondents before conducting a pilot test of the survey among a few respondents to determine its efficiency in data collection.

The data collection process involved conducting a survey via an electronic questionnaire that was designed using Google Forms platform and sent out to potential respondents through WhatsApp groups. The participants were given proper instructions about the purpose of the study and how their participation was voluntary. The questionnaire was kept open for almost 2 weeks so that students had adequate time to fill out the questionnaire. After obtaining the required data, it was filtered and prepared for statistical analysis. Descriptive statistics were calculated first to see how students responded and what patterns were observed for using self-regulated learning strategies.

It is important to mention here that before data analysis, all negatively worded items were reverse-coded to ensure that higher scores consistently represented higher levels of self-regulated learning. Reverse coding was performed using the standard transformation for five-point Likert-scale responses (1 = 5, 2 = 4, 3 = 3, 4 = 2, 5 = 1). The reverse-coded items were goal setting (Items 8 and 10), time management (Items 5 and 7), self-monitoring (Items 4 and 6), and self-evaluation (Items 3 and 4). After reverse coding, composite subscale scores were computed by averaging the ten items corresponding to each SRL construct. These validated subscale scores were subsequently used in the correlation and multiple regression analyses.

### 3.4 Validity and reliability

In order for the research instrument to be valid, a number of steps were followed. First, content validity was ensured through the use of existing research instruments. The research instrument was adapted from Barnard et al.'s (2009) OSRLQ and modified to reflect the context of virtual learning among Saudi EFL majors. Minor wording revisions were made to ensure relevance to virtual learning tasks, assignments, and learning activities. It was also aligned with other research literature on self-regulated learning and virtual learning (Zimmerman, 2002; Pintrich, 2004). Experts were consulted from the field of applied linguistics and educational technology to ensure that the research instrument was valid and that it could measure self-regulated learning constructs, particularly goal setting, time management, self-monitoring, and self-evaluation for virtual EFL learning. The EFL experts verified the questionnaire and revised its items. They recommended few modifications that were adequately implemented before starting the process of data collection. Furthermore, a pilot study was subsequently conducted using a small group (*n* = 20) of EFL learners before actual data collection. Also, in order for the research instrument to be reliable, Cronbach’s alpha was used. Reliability analysis indicated satisfactory internal consistency for goal setting (*α* = 0.84), time management (*α* = 0.81), self-monitoring (*α* = 0.88), and self-evaluation (*α* = 0.86). This is considered an acceptable reliability coefficient for Likert-type scales (Field, 2018). The value of alpha was considered satisfactory at values of 0.70 and above (Cohen et al., 2018). The steps that were followed ensured that the research instrument was valid and could measure the reliability of EFL learners’ use of SRL strategies (see Table 1).

**Table 1:** Validity and reliability evidence for the four SRL constructs (*N* = 270).

Construct	Source of construct	Content validity	Pilot tested	Construct validity evidence
Goal setting	Adapted from Barnard et al. (2009) OSLQ; Zimmerman (2002); Pintrich (2004)	Expert-reviewed	Yes (*n* = 20)	Measure learners’ ability to establish, plan, and regulate learning goals in VLEs
Time management	Adapted from Barnard et al. (2009) OSLQ; Zimmerman (2002); Pintrich (2004)	Expert-reviewed	Yes (*n* = 20)	Measure learners’ ability to organize, allocate, and regulate study time in VLEs
Self-monitoring	Adapted from Barnard et al. (2009) OSLQ; Zimmerman (2002); Pintrich (2004)	Expert-reviewed	Yes (*n* = 20)	Measure learners’ ability to track progress, monitor comprehension, and adjust learning strategies
Self-evaluation	Adapted from Barnard et al. (2009) OSLQ; Zimmerman (2002); Pintrich (2004)	Expert-reviewed	Yes (*n* = 20)	Measure learners’ ability to reflect upon outcomes, assess performance, and revise learning plans
General instrument	OSLQ and SRL literature	Expert-reviewed	Yes (*n* = 20)	Measures four theoretically established dimensions of self-regulated learning

The results from the reliability analysis in Table 2 indicate that all four self-regulation constructs had high internal consistencies in terms of their Cronbach’s alpha coefficients (0.81–0.88). Self-monitoring recorded the highest reliability coefficient (0.88), followed by self-evaluation (0.86), goal setting (0.84), and time management (0.81). Reliability coefficient for the whole questionnaire was 0.85, which is considered good (Field, 2018). As all alphas were above the required 0.70, the reliability test indicated that the questionnaire had sufficient reliability to measure self-regulation constructs among Saudi EFL students in VLEs.

**Table 2:** Reliability analysis of the measurement instrument (*N* = 270).

Construct	No. of items	Cronbach’s alpha (*α*)
Goal setting	10	0.84
Time management	10	0.81
Self-monitoring	10	0.88
Self-evaluation	10	0.86
General instrument	40	0.85

Furthermore, subscale scores were computed by averaging the responses to the ten items associated with each self-regulated learning dimension. Prior to score computation, all negatively worded items were reverse-coded so that higher scores consistently represented higher levels of self-regulated learning. The resulting composite scores demonstrated satisfactory internal consistency, with Cronbach’s alpha coefficients ranging from 0.81 to 0.88. These validated subscale scores were subsequently used in the descriptive, correlational, and regression analyses.

### 3.5 Data analysis

The data analysis was carried out using Statistical Package for the Social Sciences (SPSS) (Field, 2018; Pallant, 2020) to explore the relationship between SRL strategies and academic performance of Saudi EFL learners in VLEs. The first stage was descriptive statistics, which were computed to understand how SRL strategies were being adopted by the learners. The study collected data on four aspects of SRL strategies: goal setting, time management, self-monitoring, and self-evaluation. The next stage was to carry out Pearson correlation analysis to show the extent to which SRL strategies were linked with academic performance. Finally, multiple regression analysis was conducted to examine the predictive power of SRL strategies on Saudi EFL majors’ academic performance in virtual EFL contexts.

### 3.6 Research procedures

The research procedures involved several steps to collect and analyze the data accurately. To begin with, ethical clearance was sought from the relevant academic department, and the participants were made aware of the purpose of the study, the voluntary nature of the research participation, and the confidentiality of the data collected. After obtaining the consent of the subjects, the questionnaire designed to measure the level of the four SRL strategies was sent to the participants electronically through WhatsApp groups. The participants were clearly instructed on how to complete the questionnaire correctly, and they were given about 2 weeks to complete the questionnaire. After obtaining the data from the respondent participants, the accuracy of the data collected was examined by filtering out the incomplete questionnaires. The data collected were then encoded and entered into SPSS to analyze the data accurately. The data were first analyzed to find out the level of SRL strategies used by Saudi EFL students in VLEs. Then, correlation and regression tests were employed to show the relationships between the four variables of SRL and students’ academic performance in virtual EFL courses.

Moreover, the ethical conduct principles were adhered to throughout the research process. Prior to beginning the data collection process, the participants were informed about the nature of the research, its implications, and their rights not to take part in the survey if they wish to do so at any stage without being penalized in any way. Consent was obtained online before answering the survey. Confidentiality and anonymity of the data were ensured as there was no requirement for personal information to be revealed. The data was collected for scholarly purposes as suggested in the literature on conducting academic research (Cohen et al., 2018; Creswell and Creswell, 2018).

## 4 Analysis and results

This section presents the results obtained from the statistical analyses that show the connection between SRL strategies and academic performance of Saudi EFL students in VLEs.

### 4.1 Results pertaining to the demographic data of the participants

As mentioned before, the sample of the current study is 270 Saudi EFL undergraduate students, who are all enrolled in virtual English language courses. Data related to demographics were gathered regarding their gender, age, nationality, and educational levels (see Table 3).

**Table 3:** Demographic characteristics of the participants (*N* = 270).

Variable	Category	No.	(%)	Total
Gender	Male	165	61.1	
	Female	105	38.9	270
Age	17–18	38	14.1	
	19–20	112	41.5	
	21–22	86	31.9	
	23	34	12.6	270
Nationality	Saudi	270	100	270
Academic level	Level 1	32	11.9	
	Level 2	35	13.0	
	Level 3	34	12.6	
	Level 4	36	13.3	
	Level 5	33	12.2	
	Level 6	34	12.6	
	Level 7	33	12.2	
	Level 8	33	12.2	270

As indicated from Table 3, the participants involved in the study included 270 EFL learners who were Saudi students only to ensure cultural homogeneity among the respondents. As far as gender is concerned, 165 out of 270 participants were male (61.1%), whereas the remaining 105 respondents were female (38.9%). In terms of age, it was observed that the majority of the participants (41.5%) were aged between 19 and 20 years, while those aged between 21 and 22 years were (31.9%), which is a common age group for EFL students in Saudi Arabia. Furthermore, those aged between 17 and 18 years were also present in the study, while those aged 23 years were only (12.6%), which could indicate those in the advanced level of their studies. In terms of their academic level, it was observed that all levels, ranging from 1 to 8, were represented in almost equal numbers, with each level being around (12–13%) of the total participants. This indicates that all levels were represented in the study, ensuring that it was a fair representation in terms of how SRL constructs are significant correlates of Saudi EFL majors’ academic performance.

### 4.2 Results pertaining to descriptive statistics

Descriptive statistics were also employed to gain an idea of the extent to which the four SRL strategies differed from one participant to another. The findings indicate that students rated goal setting and self-evaluation moderately, but time management was slightly behind the rest in terms of the averages. From the data on the performance of the students, it was evident that the differences in the performance of the students in the virtual EFL courses were moderate.

Table 4 clarifies that Saudi EFL students generally show moderate to high encouragement with self-regulated learning strategies in VLEs. However, the comparatively lower mean score for time management indicates that some students encounter some difficulties when they attempt to organize and manage their study time efficiently in virtual contexts.

**Table 4:** Descriptive statistics for SRL variables and academic performance (*N* = 270).

SRL constructs	Mean	SD	Min	Max
Goal setting	3.86	0.61	2.00	5.00
Time management	3.54	0.69	1.90	4.90
Self-monitoring	3.79	0.63	2.10	5.00
Self-evaluation	3.91	0.58	2.30	5.00
Academic performance (GPA)	3.45	0.46	2.00	4.00

The validated subscale scores in Table 5 indicate that Saudi EFL learners reported relatively high levels of self-regulated learning across all four dimensions. Goal setting obtained a composite mean of 3.86 (*α* = 0.84), suggesting that students generally established clear learning objectives and monitored their progress toward achieving them. Self-monitoring recorded a similarly high score (M = 3.79, *α* = 0.88), indicating that learners frequently tracked their understanding, identified errors, and adjusted their learning strategies when necessary. Self-evaluation also demonstrated a high level of endorsement (M = 3.91, *α* = 0.86), reflecting learners’ tendency to assess their learning outcomes and make appropriate adjustments to their learning plans. Time management achieved the lowest, although still relatively positive, composite mean (M = 3.54, *α* = 0.81), suggesting that students were somewhat less consistent in organizing and allocating study time effectively compared to the other SRL dimensions. The reliability ranges of values between 0.81 and 0.88 are indicative of strong internal consistency for all the four sub-scales, thus implying that the questionnaire is relevant to measure the desired concepts pertaining to SRL in an effective manner. The results confirm the validity and reliability of the four SRL sub-scales for further correlation and regression analysis related to virtual academic performance.

**Table 5:** Validated subscale scores of the SRL questionnaire (*N* = 270).

SRL construct	No. of items	Cronbach’s *α*	Mean	SD
Goal setting	10	0.84	3.86	0.61
Time management	10	0.81	3.54	0.69
Self-monitoring	10	0.88	3.79	0.63
Self-evaluation	10	0.86	3.91	0.58

### 4.3 Results pertaining to correlation analysis

Pearson correlation analysis was employed to examine the relationship between SRL variables and students’ academic performance. The results show that all SRL strategies had significant positive correlations with students’ academic performance in virtual EFL courses.

The correlation analysis in Table 6 indicated that there are moderately positive correlations among the four constructs of self-regulated learning, with correlation coefficients varying between 0.42 and 0.58. The highest correlation coefficient was found between self-monitoring and self-evaluation (*r* = 0.58, *p* < 0.01), whereas the lowest correlation was identified between time management and self-evaluation (*r* = 0.42, *p* < 0.01). While there were connections between the variables, their correlation did not reach the level that could be considered indicative of the presence of multicollinearity problems (*r* ≥ 0.80). This means that the four variables reflect different aspects of self-regulated learning.

**Table 6:** Correlation matrix for SRL variables and academic performance.

Variables	1	2	3	4	5
1. Goal setting	—				
2. Time management	0.50**	—			
3. Self-monitoring	0.54**	0.47**	—		
4. Self-evaluation	0.49**	0.42**	0.58**	—	
5. Academic performance	0.44**	0.38**	0.48**	0.46**	—

*p* < 0.01.

### 4.4 Results pertaining to multiple regression analysis

A multiple regression analysis was implemented to decide the predictive power of the four SRL strategies on students’ academic performance. The regression model was statistically significant and revealed that SRL strategies together are significant predictors to explaining variation in academic achievement.

The results of the regression analysis in Table 7 indicate that all SRL constructs account for variance (41%) in academic performance. When we examine each predictor separately, self-monitoring is found to be the most significant predictor with a beta value of (0.31). The next three predictors are self-evaluation with a beta value of (0.27), goal setting with (0.25), and time management with (0.21). Significantly, it can be noticed that students who self-monitor their learning tend to achieve better academic performance in VLEs.

**Table 7:** Multiple regression analysis predicting academic performance.

Predictor	B	SE B	*β*	*t*	*p*
Goal setting	0.22	0.06	0.25	3.67	<0.001
Time management	0.18	0.05	0.21	3.21	0.001
Self-monitoring	0.27	0.07	0.31	3.94	<0.001
Self-evaluation	0.24	0.06	0.27	3.73	<0.001

*R*^2^ = 0.41, Adjusted *R*^2^ = 0.40, *F*(4,265) = 45.83, *p* < 0.001.

The multicollinearity diagnostics in Table 8 demonstrate that the regression model met the assumption of predictor independence. Tolerance values ranged from 0.56 to 0.68, exceeding the recommended minimum threshold of 0.20, while VIF values ranged from 1.47 to 1.79, well below the critical value of 5.00. Although the SRL predictors were moderately correlated (*r* = 0.42–0.58), the diagnostic statistics indicate that the degree of shared variance among the predictors was not sufficiently high to threaten the stability of the regression estimates. Consequently, goal setting, time management, self-monitoring, and self-evaluation were retained in the regression model, and the regression coefficients can be interpreted as representing the significant association of each SRL construct with academic performance.

**Table 8:** Multicollinearity diagnostics for the SRL predictors of academic performance (*N* = 270).

Predictor	Tolerance	VIF
Goal setting	0.63	1.59
Time management	0.68	1.47
Self-monitoring	0.56	1.79
Self-evaluation	0.61	1.64

### 4.5 Results pertaining to students’ questionnaire

This part of the analysis constitutes four subsections that present the perception of Saudi EFL students about the extent to which self-regulated learning constructs are associated with their academic performance within virtual learning environments.

The results pertaining to the questionnaire on the SRL construct of goal setting (see Supplementary Table S1) demonstrate that participants tend to set goals at a high level because the average score of the positive items ranges from (M = 3.82) to (M = 4.01). This shows consistent agreement and high engagement in effective strategic learning behaviors such as planning, prioritizing, and monitoring goals. More than 60% of the participants’ answers are categorized as agreement or strong agreement, which mirrors the positive attitude of the respondent participants. On the other hand, the negative items (items 8 and 10) indicate significant disagreement with more than (60%) of the participants strongly disagreeing or disagreeing with the items and having relatively low average scores ranging from (M = 2.44) to (M = 2.46). This, in turn, clarifies that the participants reject ineffective goal-setting behaviors. The relatively larger range of the average score shows some level of inconsistency among participants. The results thus demonstrate the participants’ consistent and effective self-regulated goal-setting behaviors, which supports the construct validity of the scale.

Concerning the questionnaire on the SRL construct of time management (see Supplementary Table S2), the obtained results indicate that there is a positive attitude in terms of time management skill usage by the participants. The results show that all the average values of the items range from (M = 3.46) to (M = 3.65). This shows that nearly all the participants agree that good time management and planning are an essential part of their life. The majority of the results show that all the participants agree (45–50%) with the statements. For items 5 and 7, which were negatively phrased, there is strong evidence of disagreement from all the participants, as shown by more than half of the participants using strongly disagree or disagree and low average values of (M = 2.68) and (M = 2.69). This shows that all the participants disagree with bad time management and tracking. However, item 6, which asks about how students manage their time for studying, shows low values (M = 3.46). This indicates that participants experience some difficulties in being consistent across all situations.

In terms of self-monitoring questionnaire (see Supplementary Table S3), results suggest that respondent students generally have a positive perception of self-monitoring. The range of the average rating of the positive items falls between (M = 3.80) and (M = 3.92), implying that students frequently make use of metacognitive strategies such as monitoring of progress, detection of errors, and evaluation of performance. Nearly half of the students’ responses to each of the items fall under “agree,” implying that students recurrently make use of these strategies to regulate their understanding and learning processes. For the negative items (items 4 and 6), students generally disagree with the ineffective strategies of self-monitoring, as indicated by at least half of the students’ responses falling under “strongly disagree” and “disagree” and the range of the average rating falling between (M = 2.66) and (M = 2.68).

As for the SRL construct of self-evaluation (see Supplementary Table S4), the obtained results show that participants are always engaged in self-evaluation processes. The average score of the positive items ranges from (M = 3.80) to (M = 3.94), which shows the continued engagement of participants in self-reflection and evaluation processes such as checking one’s performance, reassessing one’s goals, and adjusting one’s study plan. Moreover, the tendency of the participants’ answers towards the “agree” signifies that the participants are always engaged in self-reflection and evaluation processes by stopping and reflecting on the way they are performing or approaching the tasks. On the other hand, the negatively phrased items (3 and 4) indicate that more than half of the participants’ answers are strongly inclined towards the “strongly disagree” and “disagree” options with average ratings ranging from (M = 2.66) to (M = 2.68), which shows that the participants strongly reject ineffective self-evaluation processes. These results indicate that the participants are highly inclined towards self-reflection and evaluation processes, which, in turn, demonstrates the solid role of evaluative processes in the self-regulated learning of the participants.

## 5 Discussion

The aforementioned results show that the four SRL constructs, namely goal-setting, time management, self-monitoring, and self-evaluation, are significant predictors of academic performance among Saudi EFL majors in virtual learning settings. Descriptive analysis results, on the one hand, indicate that SRL strategies are employed differently, with self-monitoring and self-evaluation achieving the highest averages. These results suggest that Saudi EFL students are most likely engaging in metacognitive thinking. Correlational analysis results, on the other hand, demonstrate that SRL strategies are positively related to academic performance, with self-monitoring strategy achieving the highest correlation coefficient (*r* = 0.48). These findings are consistent with Zimmerman (2002) framework that SRL strategies are important tools that learners use in their academic planning and evaluation and also correlate with previous empirical studies (e.g., Barnard et al., 2009; Broadbent and Poon, 2015; Panadero, 2017; Simón-Grábalos et al., 2025), which accentuate that students who are able to efficiently self-regulate their learning show better levels of academic performance. This study, therefore, provides evidence that SRL strategies are significant correlates of academic performance in the Saudi virtual EFL contexts.

The analysis of the collected data demonstrate that the exceptional average score of goal setting by the Saudi EFL students, with a mean of (3.86), indicates that respondent students readily engage in defining their learning goals, prioritizing tasks, and outlining their learning plan for the virtual courses they take. This finding reconciles with Alanazi (2020); Almayez et al. (2025); and Zheng and Sun’s, 2025) arguments that goal setting is the key motivator that helps students become focused, as this helps them utilize their resources efficiently, which is the basis of the self-regulated learning process that students engage in at a later time, as they engage in the process of monitoring and evaluating their own learning. However, despite the high engagement of the Saudi EFL students in the goal-setting process, it was noted that these students were not as consistent in documenting their learning goals. This indicates that respondent students entirely depend on their cognitive abilities when engaging in the goal-setting process, as opposed to the written strategies that they could employ in the process. Results also show that students may benefit from structured goal-setting practices, which goes in conformity with Broadbent and Poon’s (2015) argument that learners are more likely to engage with educational expectations when they explicitly formulate and document their learning goals. This, in turn, indicates that students benefit from the goal-setting process if the virtual courses they take are designed with the goal-setting feature. Such a goal-setting process is the key motivator that is positively associated with Saudi EFL majors’ academic performance in VLEs.

Concerning time management, it is analytically evidenced that it receives a moderate average rating of (3.54), signifying that respondent students attempt to set aside dedicated study time and adhere to deadlines; however, the ability to adhere to these learning practices is not as strong as with other SRL strategies. This finding reconciles with some previous studies (e.g., Barnard et al., 2009; Abate et al., 2025; Alhroob and Al-khresheh, 2026), which emphasize that time management is the least consistently utilized aspect of self-regulated learning. In the Saudi EFL context, the domestic duties, co-curricular activities, and level of technological competence may be associated with students’ ability to establish and maintain effective time-management practices for learning. However, the correlation with academic achievements is still (0.38), which means that although time management plays an essential role, it is not as much of a determining factor of academic performance as meta-cognitive learning skills like self-regulation. The findings, therefore, suggest that the quality and focus of learners’ study practices may be more closely associated with learning outcomes than the amount of time spent studying within virtual EFL learning environments.

Regarding self-monitoring, the analysis shows that it proves to be the most important predictor of academic performance among other SRL strategies (*r* = 0.48). Self-monitoring means that students are likely to check their comprehension of information, identify mistakes, and make corrections if needed. It can be explained by the fact that this factor coincides with Zimmerman (2002) SRL model which considers metacognitive monitoring to be the heart of self-regulated learning, especially when there is no feedback. Furthermore, this factor also matches with the assertion made by Panadero (2017) which states that self-regulated learners who check their understanding of information will more easily regulate their strategies and avoid making any mistakes. As for virtual EFL learning, the importance of self-monitoring cannot be overestimated because it is mostly asynchronous, thus forcing students to control their learning process on their own. The reliability coefficient of the self-monitoring scale was relatively high (*α* = 0.70). Thus, the assumption that Saudi EFL learners prefer using reflective strategies to regulate learning processes is valid.

As for self-evaluation, the analysis demonstrates that this strategy is characterized by a high mean (M = 3.91), similar to what is observed when Saudi EFL majors reflect on their task accomplishment, assess their goal fulfillment, and adjust their strategies based on their observations. This finding aligns with the assumptions revealed by some previous studies (e.g., Zhao et al., 2025; Zheng and Sun, 2025; Waheed et al., 2025), who maintain that self-evaluation is an integration of learning and enables learners to plan their actions in the future better. Significantly, the analysis further highlights that Saudi EFL majors solely rely on their self-regulatory reflection and do not receive any external feedback; this trend is typical of the Saudi culture educational practices that prioritize self-sufficiency (Almayez et al., 2025). In light of this observation, it becomes evident that virtual learning platforms have an essential role to play in promoting self-evaluation by implementing reflection cues, peer evaluation chances, and feedback systems. As a result, instructors are expected to encourage learners to convert self-reflection into a learning strategy that may affect their subsequent actions.

The obtained results further reveal the strong interrelation between the SRL strategies, with correlations, which highlights the reciprocal support of these strategies. In other words, the implementation of goal-setting helps with the process of monitoring, which, in turn, helps with the process of evaluation, creating a feedback effect that increases self-regulation. This is in accordance with Zimmerman (2002) SRL framework, which is also supported by the findings of the studies in the context of CALL (e.g., Locke and Latham, 2002; Broadbent and Poon, 2015). The interrelation between the SRL constructs means that, if changes are made in one of the variables, this could have a spill-over effect on the other variables as well. In other words, if goal-setting is integrated, this could improve the students’ ability in the variables of monitoring and evaluation, which could, in turn, improve their academic performance. As such, the interrelation between SRL constructs emphasizes the necessity of scaffolding the entire range of SRL strategies, as opposed to focusing on individual strategies, in virtual EFL courses.

Finally, the findings of this study move beyond a simple demonstration that SRL correlates with academic performance towards examining how distinct SRL constructs function within a Saudi online EFL context, where learners often navigate a transition from more teacher-directed educational experiences to environments that require greater learner autonomy. Although there is a significant relationship between the four SRL strategies and Saudi EFL majors’ academic performance, such a correlation may not be generalized to other educational settings. This is because of the Saudi context-oriented nature of the finding of this study. As such, the extent to which the findings of this study are transferable beyond the Saudi EFL context may depend on contextual factors such as culture, educational practices, technological access, and learning environment characteristics. The findings indicate that self-monitoring emerged as the strongest predictor of academic performance, suggesting that performance-phase regulation may be particularly critical in VLEs where immediate instructor guidance is less available. This result refines existing applications of Zimmerman (2002) cyclical model by highlighting that the relative importance of SRL dimensions may vary across cultural, linguistic, and technological contexts. In this way, the study offers context-sensitive evidence that contributes to a much more understanding of how SRL operates in virtual EFL learning rather than simply replicating previously established SRL–achievement relationships.

## 6 Conclusion

This study was carried out in order to examine the relationship between four SRL strategies, including goal-setting, time management, self-monitoring, and self-evaluation, and the academic achievement of Saudi EFL learners in their virtual English classes. It was analytically evidenced that the four strategies showed a significant positive relationship with the academic achievement of Saudi EFL learners. The analysis also highlighted that metacognitive strategies are crucial in enabling students to learn on their own and successfully in a virtual world, thus supporting Zimmerman (1989) theory of self-regulated learning. The analysis further demonstrated that, although goal setting and time management are consistently used, their relationship with academic performance is only moderate in comparison to self-monitoring and self-evaluation. This indicates that in EFL asynchronous virtual learning, it is crucial for students to monitor their own progress and thoughtfully evaluate their results in order to improve their academic performance, rather than entirely depending on goal setting. It was also clarified that there are certain factors, such as the traditional teacher-centered teaching style, which might have significant relationships with the application of formal goal setting and time management strategies by Saudi EFL students.

## 7 Implications

This study has some implications for EFL instructors, curriculum designers, and virtual learning practitioners in Saudi higher education and similar contexts. First, the significant association of SRL constructs with academic performance suggests that metacognitive scaffolding should be embedded within virtual language courses. It would be practical for educators to come up with activities that enable learners to monitor their understanding of information, assess how far they have gone, and evaluate themselves to ensure continuous engagement and independence in their learning process. The use of such strategies as reflective questions, progress dashboard, and self-assessment rubrics is anticipated to assist in accomplishing these strategies using technology-based tools.

Second, concerning curriculum development in EFL and virtual learning programs, the study recommends that courses should explicitly teach SRL strategies, incorporating structured modules on goal setting, time management, self-monitoring, and self-evaluation. Embedding strategy instruction alongside language content can cultivate learners’ strategic competence, which, in turn, functions to bridge the gap between language acquisition and autonomous learning. Moreover, cultural considerations are essential; Saudi learners may benefit from scaffolded guidance that gradually shifts responsibility from instructors to students, aligning with the findings that goal setting and time management were less consistently applied than metacognitive strategies.

Third, the importance of self-regulated learning (SRL) in virtual learning should be emphasized in teacher preparation programs. Instructors should be equipped with not only pedagogical knowledge but also technical skills in the creation of learning environments that support SRL. Consequently, instructors should be trained in monitoring students’ engagement, facilitating students in setting their goals, and supporting students in their self-reflection and evaluation. By enhancing instructors’ capacity in facilitating SRL in instruction, students are more likely to develop SRL skills and improve their academic performance.

## 8 Limitations and future research

This study has a number of limitations as follows: First, the study focused only on Saudi EFL students, with a sample size of 270 students from only one university. The limited scope of the study indicates that the findings of this study is context-bound and cannot be generalized and transferred beyond the Saudi EFL context. Such findings transferability beyond the Saudi EFL context depends on various contextual factors, including culture, technological resources availability, and learning strategies. Second, the study primarily depends on self-report questionnaires as a method for measuring the level of students’ use of SRL strategy, which suggests that it can be affected by social desirability, memory, or participants’ tendency to overestimate their engagement in SRL practices. Third, the study examined only four aspects of self-regulated learning, namely goal setting, time management, self-monitoring, and self-evaluation, and it did not examine other aspects that could demonstrate important correlations with academic performance, such as motivation, language proficiency, and computer literacy, as well as cognitive and emotional load. Consequently, some of the variance observed in the findings may reflect factors other than SRL.

As for future research, the current study proposes a possible area for further investigation into the topic of SRL in other virtual EFL settings. The issues that need to be explored in future work include the dynamics of SRL intervention implementation, the connection between SRL and motivation and language proficiency, and the strategies used in various cultures. A combination of qualitative and quantitative techniques, for example the analysis of learner reports along with LMS logs, can lead to more detailed insights about the topic under investigation. This may contribute to the evidence-based development of CALL technology, enhance SRL, and continue to develop SRL theory in different educational settings.

## Data Availability

The original contributions presented in the study are included in the article/Supplementary material, further inquiries can be directed to the corresponding author.
