# Supplementary material for: Self-regulated learning constructs as predictors of academic performance among Saudi EFL students in virtual learning environments: Insights from self-regulated learning theory
Source: Front Psychol. 2026 Jul 21;17:1859492. doi: 10.3389/fpsyg.2026.1859492 (PMC13433730; doi:10.3389/fpsyg.2026.1859492)
Supplement: Supplementary file 1 [file Data_Sheet_1.pdf]

Appendix

TABLE S1 Saudi EFL students' perceptions of SRL construct of goal setting as predictor of academic performance within VLEs

No.	Item	SD		D		Neutral		A		SA		Mean	SD
		(No.)	(%)	(No.)	(%)	(No.)	(%)	(No.)	(%)	(No.)	(%)		
**1**	I set specific goals for each virtual learning session.	12	4.4%	18	6.7%	45	16.7%	120	44.4%	75	27.8%	4.01	0.91
**2**	I prioritize my language learning tasks according to their importance.	9	3.3%	21	7.8%	42	15.6%	126	46.7%	72	26.7%	4.01	0.88
**3**	I break large learning tasks into smaller, manageable goals.	15	5.6%	18	6.7%	48	17.8%	126	46.7%	63	23.3%	3.92	0.93
**4**	I set clear objectives before starting virtual assignments.	12	4.4%	24	8.9%	51	18.9%	129	47.8%	54	20.0%	3.88	0.89
**5**	I review my goals regularly to stay on track.	9	3.3%	27	10.0%	54	20.0%	132	48.9%	48	17.8%	3.85	0.88
**6**	I focus on achieving both short-term and long-term learning goals.	12	4.4%	24	8.9%	60	22.2%	132	48.9%	42	15.6%	3.82	0.90
**7**	I adjust my goals when encountering difficulties.	18	6.7%	27	10.0%	54	20.0%	123	45.6%	48	17.8%	3.82	0.95
**8**	I do not write down my learning goals for each course. *(Negative)*	78	28.9%	81	30.0%	45	16.7%	39	14.4%	27	10.0%	2.46	1.21
**9**	I set realistic goals based on my current abilities.	15	5.6%	24	8.9%	60	22.2%	132	48.9%	39	14.4%	3.82	0.91
**10**	I rarely use my goals to motivate myself to complete tasks. *(Negative)*	81	30.0%	84	31.1%	39	14.4%	36	13.3%	30	11.1%	2.44	1.23

Scale: SD = Strongly Disagree, D = Disagree, N = Neutral, A = Agree, SA = Strongly Agree

TABLE S2 Saudi EFL students' perceptions of SRL construct of time management as predictor of academic performance within VLEs

No.	Item	SD		D		Neutral		A		SA		Mean	SD
		(No.)	(%)	(No.)	(%)	(No.)	(%)	(No.)	(%)	(No.)	(%)		
**1**	I schedule specific times for studying English virtually.	18	6.7%	30	11.1%	63	23.3%	129	47.8%	30	11.1%	3.63	0.96
**2**	I avoid procrastination when completing virtual tasks.	15	5.6%	36	13.3%	60	22.2%	135	50.0%	24	8.9%	3.57	0.93
**3**	I allocate enough time for reading, writing, listening, and speaking practice.	12	4.4%	30	11.1%	69	25.6%	132	48.9%	27	10.0%	3.65	0.91
**4**	I follow a daily or weekly study plan for virtual courses.	18	6.7%	36	13.3%	66	24.4%	123	45.6%	27	10.0%	3.58	0.96
**5**	I often delay starting assignments until the last minute. *(Negative)*	60	22.2%	75	27.8%	54	20.0%	51	18.9%	30	11.1%	2.69	1.18
**6**	I balance my study time with other personal activities.	24	8.9%	39	14.4%	69	25.6%	114	42.2%	24	8.9%	3.46	0.98
**7**	I do not keep track of the time spent on virtual learning tasks. *(Negative)*	66	24.4%	72	26.7%	48	17.8%	51	18.9%	33	12.2%	2.68	1.20
**8**	I plan my study schedule	18	6.7%	39	14.4%	63	23.3%	123	45.6%	27	10.0%	3.58	0.95

	to avoid last-minute cramming.												
**9**	I adjust my study time based on task difficulty.	24	8.9%	33	12.2%	63	23.3%	129	47.8%	21	7.8%	3.50	0.92
**10**	I use reminders or digital tools to manage my time effectively.	21	7.8%	30	11.1%	60	22.2%	132	48.9%	27	10.0%	3.61	0.92

TABLE S3 Saudi EFL students' perceptions of SRL construct of self-monitoring as predictor of academic performance within VLEs

**No.**	**Item**	**SD**		**D**		**Neutral**		**A**		**SA**		**Mean**	**SD**
		(No.)	(%)	(No.)	(%)	(No.)	(%)	(No.)	(%)	(No.)	(%)		
**1**	I check my understanding while completing virtual tasks.	12	4.4%	24	8.9%	57	21.1%	138	51.1%	39	14.4%	3.87	0.88
**2**	I track my progress toward learning goals regularly.	9	3.3%	24	8.9%	54	20.0%	144	53.3%	39	14.4%	3.92	0.87
**3**	I identify errors or mistakes in my language tasks.	12	4.4%	27	10.0%	60	22.2%	135	50.0%	36	13.3%	3.85	0.89
**4**	I do not compare my performance with previous attempts.	63	23.3%	72	26.7%	54	20.0%	51	18.9%	30	11.1%	2.68	1.19
**5**	I notice which strategies help me learn best.	12	4.4%	27	10.0%	57	21.1%	135	50.0%	39	14.4%	3.87	0.88
**6**	I do not monitor my comprehension while reading or listening.	66	24.4%	75	27.8%	48	17.8%	48	17.8%	33	12.2%	2.66	1.21
**7**	I adjust my approach when I realize I am not understanding.	15	5.6%	30	11.1%	57	21.1%	135	50.0%	33	12.2%	3.80	0.90
**8**	I reflect on my performance after completing tasks.	12	4.4%	27	10.0%	60	22.2%	138	51.1%	33	12.2%	3.82	0.88
**9**	I identify gaps in my knowledge independently.	15	5.6%	30	11.1%	57	21.1%	132	48.9%	36	13.3%	3.83	0.90
**10**	I seek to correct mistakes after self-assessment.	12	4.4%	27	10.0%	60	22.2%	135	50.0%	36	13.3%	3.84	0.89

TABLE S4 Saudi EFL students' perceptions of SRL construct of self-evaluation as predictor of academic performance within VLEs

**No.**	**Item**	**SD**		**D**		**Neutral**		**A**		**SA**		**Mean**	**SD**
		(No.)	(%)	(No.)	(%)	(No.)	(%)	(No.)	(%)	(No.)	(%)		
**1**	I reflect on my performance after completing virtual tasks.	12	4.4%	24	8.9%	54	20.0%	138	51.1%	42	15.6%	3.88	0.88
**2**	I evaluate whether my goals were achieved.	9	3.3%	21	7.8%	54	20.0%	141	52.2%	45	16.7%	3.94	0.86
**3**	I do not assess my learning strategies to see if they are effective.	66	24.4%	75	27.8%	48	17.8%	48	17.8%	33	12.2%	2.66	1.21
**4**	I do not compare my results with expected outcomes.	63	23.3%	72	26.7%	54	20.0%	51	18.9%	30	11.1%	2.68	1.19
**5**	I reflect on what I did well and what I need to improve.	9	3.3%	24	8.9%	63	23.3%	141	52.2%	33	12.2%	3.80	0.86
**6**	I seek feedback and incorporate it into my learning.	15	5.6%	30	11.1%	57	21.1%	132	48.9%	36	13.3%	3.82	0.89
**7**	I evaluate my	12	4.4%	27	10.0%	60	22.2%	138	51.1%	33	12.2%	3.82	0.88

	progress toward long-term learning objectives.											
**8**	I judge whether my learning strategies match my goals.	15	5.6%	30	11.1%	57	21.1%	135	50.0%	33	12.2%	3.81	0.89
**9**	I identify which aspects of my learning need improvement.	12	4.4%	27	10.0%	60	22.2%	135	50.0%	36	13.3%	3.84	0.88
**10**	I adjust my learning plans based on self-evaluation.	15	5.6%	30	11.1%	57	21.1%	135	50.0%	33	12.2%	3.81	0.89
